# Integrating Sensory Perception and Wearable Monitoring to Promote Healthy Aging: A New Frontier in Nutritional Personalization

**DOI:** 10.3390/nu18020214

**Published:** 2026-01-09

**Authors:** Alessandro Tonacci, Francesca Gorini, Francesco Sansone, Francesca Venturi

**Affiliations:** 1 Institute of Clinical Physiology, National Research Council, 56124 Pisa, Italy; francesca-gorini@cnr.it (F.G.); francesco.sansone@cnr.it (F.S.); 2 Department of Agriculture, Food and Environment, University of Pisa, 56124 Pisa, Italy; francesca.venturi@unipi.it

**Keywords:** digital phenotyping, healthy aging, metabolic adaptation, nutritional adherence, personalized nutrition, sensory decline, sensory perception, wearable technologies

## Abstract

Aging involves progressive changes in sensory perception, appetite regulation, and metabolic flexibility, which together affect dietary intake, nutrient adequacy, and health-related outcomes. Meanwhile, current wearable technologies allow continuous, minimally invasive monitoring of physiological and behavioral markers relevant to metabolic health, such as physical activity, sleep, heart rate variability, glycemic patterns, and so forth. However, digital nutrition approaches have largely focused on physiological signals while underutilizing the sensory dimensions of eating—taste, smell, texture, and hedonic response—that strongly drive dietary intake and adherence. This narrative review synthesizes evidence on the following: (1) age-related sensory changes and their nutritional consequences, (2) metabolic adaptation and markers of resilience in older adults, and (3) current and emerging wearable technologies applicable to nutritional personalization. Following this, we propose an integrative framework linking subjective (implicit) sensory perception and objective (explicit) wearable-derived physiological responses into adaptive feedback loops to support personalized dietary strategies for healthy aging. In this light, we discuss practical applications, technological and methodological challenges, ethical considerations, and research priorities to validate and implement sensory–physiological integrated models. Merging together sensory science and wearable monitoring has the potential to enhance adherence, preserve nutritional status, and bolster metabolic resilience in aging populations, moving nutrition from one-size-fits-all prescriptions toward dynamic, person-centered, sensory-aware interventions.

## 1. Introduction

The global trend towards population aging [1,2] carries a number of new, unprecedented challenges in terms of, for example, the sustainability of the expenses of health systems [2,3]; despite this, it also comes along with the possibility to increase the quality of life throughout the human lifespan thanks to the development of affordable, user-friendly consumer technologies [4]. In terms of attempting to improve the standard of living of elderly people, preserving cardiometabolic health [5,6,7] and functional capability [8,9,10,11]—all factors related to quality of life—in these individuals largely and critically depends on nutrition [12,13,14]. In fact, adequate energy and protein intake, micronutrient sufficiency, and dietary patterns that support metabolic flexibility and prevent sarcopenia and frailty could modulate the overall quality of life and life expectancy on a large scale, especially among the elderly [14].

From a research perspective, one of the most intriguing aspects of aging is the associated sensory decline [15,16,17] at a level that, if its magnitude reaches significant peaks, can be considered as a real biomarker for dementia and neurodegeneration, particularly when it comes to chemical senses (i.e., olfaction and taste) which seem to be highly predictive for such phenomena [18]. This modulation can be highly interrelated with changes in appetite regulation [19], undermining dietary intake and food enjoyment and leading to reduced variety in feeding and increased preference for energetic and highly flavored foods [20,21]; this in turn can lead to nutritional imbalances, often with dramatic, life-threatening consequences for the affected individual [22]. On the other hand, correct nutritional intake significantly increases the possibility for humans to reach older ages, with an overall increase in the quality of life quality even among centenarians [23].

As highlighted in a recent review by Shariati et al. [24], older adults are becoming an increasingly relevant target group for the food sector. Within this framework, the development of food products tailored to the needs of elderly consumers poses notable challenges for both industry stakeholders and society at large [25]. Sensory evaluation protocols designed for this population should be simplified, for instance, through the use of visual or auditory scales and rapid techniques such as check-all-that-apply (CATA) in order to account for sensory limitations and age-related cognitive changes while maintaining data reliability. In this regard, hybrid methodologies that integrate rapid profiling techniques with conventional descriptive analysis represent a promising compromise between methodological robustness and operational efficiency. However, an important gap still remains in the literature due to the novelty of technologies introduced and the subjectivity of sensory and physiological responses, making it difficult to draw out a generalization of findings in some scenarios.

The adoption of age-adapted sensory methods, together with clear instructions, readable materials, and appropriately designed tasting procedures, can substantially enhance the quality of sensory data collected from elderly participants. Future developments in sensory evaluation are likely to be driven by technological innovation and an improved understanding of the specific needs associated with aging.

In parallel, the recent rise in the development and usage of novel consumer technology has been unprecedented, giving opportunity to the general public to take advantage of the services and functionalities they offer [4,26,27,28]. Wearable sensors and systems, for example, enable the real-time, continuous measurement of behaviors and physiological parameters, key to monitoring the nutritional habits of an individual, including physical activity and energy expenditure estimates [29,30,31], heart rate and heart rate variability (HRV) [32,33,34], sleep quantity and quality [35,36,37], continuous glucose monitoring (CGM) [38], skin temperature [39], and other biomarkers, in addition to those possibly linked with nutrition [40,41]. Mostly, such devices allow real-time data streams, in turn opening the door to the digital phenotyping of an individual’s metabolic and behavioral state with unprecedented temporal resolution.

Nevertheless, despite this significant technological progress, most digital nutrition models and interventions emphasize physiological signals and behavioral tracking [42,43], while the sensory drivers of food choice and eating behavior have often been neglected until recently, where some attention on the topic has been given in the literature [44,45]. Sensory perception oversees the hedonic and motivational aspects of eating [46] and can interact with the metabolic state bidirectionally. Therefore, the integration of sensory science with wearable physiological data, beyond unraveling the implicit, emotional responses of an individual to sensory stimulation [47,48,49,50,51] without relying on the biases typical of the classical sensory science approach [52], may enhance personalized strategies towards nutrition for older adults by aligning dietary recommendations with both the body’s metabolic needs and an individual’s sensory preferences and sensory capacity, either collected in a laboratory setting or a naturalistic scenario, such as during a meal, in the wider framework of the “p4 medicine” model applied to nutrition [53].

Under such premises, the present narrative review deals with the intersection of sensory changes in aging, metabolic adaptation, and wearable monitoring, proposing a novel, integrative framework for sensory-aware personalized nutrition aimed at promoting healthy aging. In such a framework, we will synthesize the relevant literature works, outline basic practical applications, and identify research gaps and methodological challenges that should be addressed to implement such approaches in the global context.

## 2. Review Methodology

To ensure comprehensive coverage of the scientific literature published between January 2015 and September 2025, a systematic search was conducted across major electronic bibliographic databases, including Web of Science, ScienceDirect, and PubMed. The terms included in the search were as follows: “olfaction”, “taste”, “chemical senses”, “chemosensory analysis”, “sensory analysis”, “nutrition”, “healthy aging”, “wearables”, “physiological signals”, “consumer technology”, and “healthy lifestyle”.

The literature selection process initially prioritized recently published review articles which were used to identify key themes, methodological approaches, and seminal contributions relevant to the scope of this narrative review. Subsequently, additional primary studies and earlier references were retrieved by screening the bibliographies of the selected reviews in order to broaden and deepen the contextual background of the topic.

All identified records were screened according to several eligibility criteria. Those included the publication date within the aforementioned time range, the publication language (English language papers were retained, whereas works published in other languages were discarded), the pertinence with the main focus of the research, and the inclusion of experimental data in the paper. Works with only methodological descriptions, without data supporting their claims, were excluded from the analysis. The evaluation was carried out independently by three reviewers, each responsible for one of the three thematic domains addressed in this work. In cases of disagreement, consensus was reached through structured discussion within a panel involving all four investigators contributing to the study selection process and manuscript drafting.

For each thematic area—namely (i) physiology, focusing on sensory impairment and cognitive decline in older adults; (ii) sensory analysis, addressing human–food interactions and explicit methodologies for panel testing and consumer profiling; and (iii) bioengineering, encompassing implicit physiological measures during food tasting—the primary inclusion criterion was the relevance of the study to the current state of the art discussed in this review. When articles addressed multiple influencing factors, a hierarchical selection approach was applied to retain only the sections most pertinent to the objectives of the present work.

Given the narrative nature of this review, no formal systematic review protocol (e.g., PRISMA) was applied.

## 3. Sensory Perception and the Aging Process

### 3.1. Age-Related Sensory Systems Modifications

The majority of physiological functions affecting health, mobility, and quality of life undergo a progressive decline, which is one of the main hallmarks of aging. This decline is the product of genetic, environmental, and lifestyle factors, leading to significant changes in various body systems and districts, such as the brain and musculoskeletal and cardiovascular systems. Sensory function is also affected, encompassing the deterioration of the five human senses (vision, hearing, touch, smell, and taste) [15] and impacting the ability of elderly individuals to interact with the surrounding environment, with marked consequences on daily living, communication, and social skills and relationships [54,55,56,57].

More specifically, aging has an impact on all the sensory modalities, including those allowing interaction with food, since all senses have, in different ways, an impact on nutrition. Notably, gustation (taste), olfaction (smell), somatosensory oral perception (texture, chemesthesis), vision, and also audition (e.g., the sound of crunchy foods), along with their processing at the brain level, are all involved in the multisensory interaction with edible compounds, determining its palatability or even its full avoidance in negative situations [58,59].

Among elderly individuals, the prevalence and severity of sensory dysfunction may vary individually in terms of magnitude and modalities, with common features including a reduced sensitivity to specific tastes [60] and a decrease in sensitivity to odors (i.e., an increased olfactory detection threshold) [61] and in olfactory identification capabilities [62]. Common hallmarks also include oral somatosensory changes, such as reduced tactile acuity [63], altered perception of fattiness [64] and reduced trigeminal sensitivity, in turn influencing texture preference and mouthfeel perception [65,66].

### 3.2. Sensory Modifications, Appetite, and Food Choices

As stated above, the decline in sensory capabilities, typical of the third age, is associated with a plethora of consequences regarding the relationship an individual has with food. These include, for example, the reduction in the hedonic reward obtained from food, leading some old individuals to eat less [67], since little satisfaction is associated with feeding, or to seek highly flavored foods [68] to compensate for the lack of sensations associated with the edible compounds they are consuming. These two actions can lead, respectively, to malnutrition or to the excessive intake of salt, sugar, or saturated fats [69], with negative consequences on the overall health and well-being of an individual [70,71].

These changes, notably in chemical sensory channels, i.e., smell and taste, can lead to an impairment of appetite regulation through brain areas alterations [72,73], which in turn is capable of influencing meal size and quality and the selection of macronutrients. In addition, such modifications are associated with a reduction in dietary variety and enjoyment, with negative consequences also in psychosocial terms also being reflected in the key activity of social eating [74,75,76].

### 3.3. Sensory Plasticity and Related Interventions

Sensory decline is usually progressive. As age increases, the associated sensory dysfunctions grow in a parallel fashion [76]. However, despite the irreversible nature of time, there is some evidence for plasticity and the effect that training has in such cohorts [77,78,79]. For example, in olfactory training, strategies for flavor enhancement and texture modification in a healthy manner—without carrying negative consequences for the health status of affected individuals—have shown promising results in the improvement of food perception, intake, and enjoyment of older people [78,79]. cuesch, multisensory approaches, combining the proper conveyance of visual, olfactory, gustatory, and textural cues, can enhance overall perceived palatability and intake without necessarily increasing the consumption of undesirable nutrients [80,81]. In this process, it is therefore pivotal to understand individual sensory profiles and preferences in order to tailor proper interventions maintaining the overall adequacy of nutrients, without altering the personal hedonic needs and desires [81].

### 3.4. Sensory Evaluation Methods for Food Products Targeting Elderly Population

Overall, the sensory impairments observed in elderly individuals, together with the age-related decline in cognitive abilities, highlight the need to carefully assess the feasibility of commonly used sensory evaluation methods prior to their implementation [24]. The main issues associated with sensory assessment in this population, along with the principal strengths and weaknesses of the proposed methodological approaches, are summarized in Table 1.

More specifically, optimal sensory evaluation with elderly participants requires a precautionary, assessor-centered approach. Clear and redundant communication, the use of warm-up tasks, controlled portion sizes, and appropriate palate-cleansing protocols are essential to ensure both data reliability and participant comfort. While digital tools and computerized questionnaires may be suitable for younger and autonomous elderly individuals, alternative paper-based formats or assisted data collection remain necessary for more dependent populations.

Simplified hedonic scales and rapid sensory profiling methods emerge as the most feasible and informative approaches for sensory evaluation in elderly populations. In contrast, descriptive analysis, although effective in healthy and autonomous older adults, is often limited by its high cognitive demands when applied to dependent individuals. Overall, integrated and flexible methodological frameworks are required to accommodate sensory decline, cognitive variability, and contextual factors, thereby supporting the development of nutritionally adequate and sensorially acceptable foods for aging populations.

## 4. Metabolic Adaptation in Aging

### 4.1. Physiological Changes Impacting Metabolism

In the progressive process of aging, individuals experience significant changes in terms of their body composition, including the loss of skeletal muscle mass and strength, i.e., sarcopenia [96], and an increase in fat mass, caused by decreased energy expenditure, which in turn is the result of a lower resting metabolic rate and reduced physical activity, both being associated with the increase in age-related pathologies and functional decline [97]. Later in life, humans experience reductions in resting metabolic rate, altered substrate utilization, and decreased metabolic flexibility (i.e., the ability to switch from fat to carbohydrate oxidation [98]). Nevertheless, the decrease in insulin sensitivity and the impairment of mitochondrial function, hallmarks of the aging process, also influence energy requirements and nutrient processing in those affected, predisposing the elderly to age-related metabolic diseases [99].

### 4.2. Markers of Metabolic Resilience

Metabolic resilience represents the body’s ability to recover and maintain optimal circulating levels of nutrients in response to external factors such as food intake, physical activity, or periods of fasting [100]. It is crucial during the life cycle to reduce the likelihood of encountering burdensome diseases such as obesity, diabetes, and cardiovascular disease [101], and it becomes a target for nutritional strategies directed to healthy aging promotion. Metabolic resilience can be characterized using different biomarkers that are measurable through wearable devices, including HRV, popular proxy for autonomic adaptability assessment [102,103], glycemic response through CGM, patterns of physical activity and sleep, and their interrelated relationships.

### 4.3. Interplay Between Sensory Changes and Metabolism

The decline in sensory capabilities experienced during the third age is capable of indirectly leading to impactful metabolic dysregulation through the alteration of dietary quality and meal patterns [67,68,69,70,71]. On the other hand, metabolic state possibly influences the sensory perception of an individual: in fact, fluctuations in blood glucose levels, inflammatory states, and the use of certain medications appear to be associated with a modulation in the functionality of key sensory channels like olfaction [104], even if their consequences are still debated [105]. However, the detection of such occurrences, possible through wearables and consumer technologies, is key to identifying potentially critical situations and when timely interventions are necessary, ultimately enhancing their efficacy.

## 5. Wearable Technologies in Nutrition and Health Monitoring

### 5.1. Wearables Overview and Measurement Types

Several kinds of wearable devices are currently available on the market for the monitoring of biomedical signals relevant to nutrition and metabolic health at large. These include the following:

- Consumer smartwatches for the detection of physical activity, step counts, estimated energy expenditure, heart rate (HR), heart rate variability (HRV), and sleep metrics.
- Chest straps and patches, capable of estimating cardiac measures and respiratory rate with higher accuracy.
- Continuous Glucose Monitoring (CGM) for assessing interstitial glucose trends and post-prandial responses.
- Temperature and skin conductance sensors to investigate thermoregulation and arousal/stress.
- Ingestible biosensors as emerging approaches to evaluate metabolic parameters.
- Environmental sensors.

#### 5.1.1. Consumer Smartwatches

The current market for smartwatches presents a plethora of solutions suitable for any needs end-users demand. Therefore, to shortlist such gadgets is nearly impossible and the only systematic way to do so is represented by the related market segmentation for such solutions in the Business-to-Client (B2C) section [106]. According to the best-selling Amazon products list reported in the data source, the four best-selling devices include the following:

- Apple Watch Series 8 (Apple Inc., Cupertino, CA, USA) [107];
- Samsung Galaxy Watch 5 Pro (Samsung Electronics Co., Ltd., Suwon, South Korea) [108];
- Amazfit GTR 3 Pro (Amazon.com, Inc., Seattle, WA, USA) [109];
- Fitbit Versa 4 (Google LLC, Mountain View, CA, USA) [110].

A comparison between them in terms of selling price (top retailers, updated on 20 October 2025) and main features of interest (biomedical signals recorded) is shown in Table 2.

The comparison of existing tools shows a significantly lower price for the Amazfit GTR3 Pro device, whereas the Apple Watch Series 8 appears to be the most expensive item. Slight differences are detected in terms of the functionality of the devices, with heart rate, oxygen saturation, physical activity, sleep monitoring, and respiration assessment as common features for all the tools under investigation.

#### 5.1.2. Chest Straps and Patches

Chest straps are wearable devices used to monitor physiological parameters, mainly HR, through the detection of their electrical signals during exercise or other experimental conditions where it is difficult to employ common clinical devices. Chest straps are normally placed at the chest level, with proper contact with the human skin assured through gel or simply through using the individual’s sweat. Commonly, they are interfaced with a third party (normally, a smartphone app) via Bluetooth, enabling continuous data streaming, display, and storage, normally on a cloud. Some of these devices are capable of monitoring other parameters, like respiration, for which they are usually considered more accurate and reliable, yet more complex, than smartwatches and other wrist-worn monitors.

As seen previously with smartwatches, the market for chest straps is very fragmented and several solutions are currently available. Among these, according to the well-designed review by Machado and collaborators [111], most frequently used models include:

- Polar H7 chest strap (Polar Electro Oy, Kempele, Finland) [112];
- Polar H10 cardiac belt (Polar Electro Oy, Kempele, Finland) [113];
- Polar T31 device (Polar Electro Oy, Kempele, Finland) [114];
- Zephyr Bioharness 3.0 chest strap (Medtronic plc, Galway, Republic of Ireland) [115];
- Movesense HR+ sensor (Movesense Oy, Vantaa, Finland) [116];
- Garmin HRM-Dual (Garmin, Schaffhausen, Switzerland) [117].

Among them, Polar solutions are known for their reliability and stability in measurements, although they appear to be purposely designed for fitness-related activities rather than for clinical assessment of ECG and connected features. On the other hand, the solution provided by Garmin seems to have its main advantages in its wearability and comfort, even with a lower accuracy in the estimation of HRV. Zephyr and Movesense devices are well designed for accurate measurements, although their comfort is somewhat debatable, especially for longer measurements [111].

ECG smart patches are also particularly suitable for some specific tasks that are undertaken in monitoring purposes and at clinics, such as for the detection of atrial fibrillation for which they appear to be highly specific [118] or for cardiogenic vertigo [119]. The most popular solution in this regard is the SmartCardia 7 L (SmartCardia SA, Ecublens, Switzerland), an innovative patch that offers real-time 7-Lead ECG and vitals integrated within Software-as-a-Service (SaaS) solution, approved as SCaAI patch and cloud platform in Europe (under the classification CE Class IIa) for the detection of ECG, respiration, SpO2, activity, and cloud-based arrhythmia detection [120].

#### 5.1.3. Continuous Glucose Monitors

**Figure 1:**
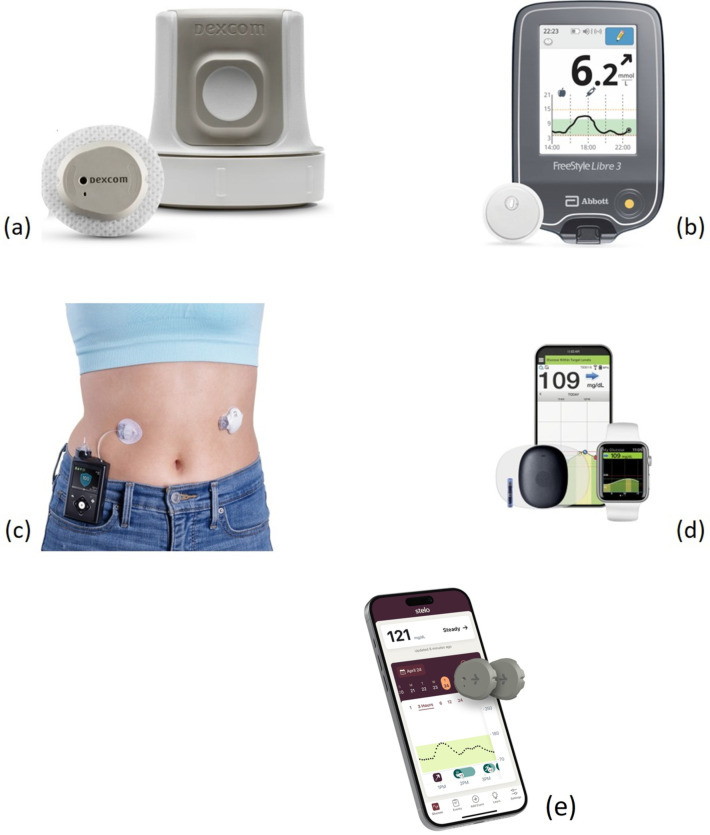
Popular commercial CGMs: (**a**) Dexcom G7 [121]; (**b**) Abbott FreeStyle Libre 3 [122]; (**c**) Medtronic Guardian 4 [123]; (**d**) Eversense 365° [124]; (**e**) Dexcom Stelo [125].

Due to the prevalence of metabolic disorders in the global population and thanks to the mounting technological developments in the field, several solutions for continuous glucose monitoring are now available on the market. Some popular choices include the following (Figure 1):

- Dexcom G7 (Dexcom, Inc., San Diego, CA, USA) [121];
- Abbott FreeStyle Libre 3 (Abbott Laboratories, North Chicago, IL, USA) [122];
- Medtronic Guardian 4 (Medtronic plc, Galway, Republic of Ireland) [123];
- Eversense 365° (Senseonics, Inc., Germantown, MD, USA) [124];
- Dexcom Stelo (Dexcom, Inc., San Diego, CA, USA) [125].

Dexcom G7 is considered among the best solutions for CGM in terms of accuracy and ease of use. The disposable sensor duration for continuous measurement is up to 10 days and communication with a third party (e.g., Apple Watch) is made possible thanks to Bluetooth Low Energy (BLE). The signal receiver has a battery life of one week and can be fully charged within 3 h.

The FreeStyle Libre 3 device is probably the most popular solution on the market and has been a global leader for many years. It distinguishes itself for its availability and cost affordability. The sensor can be worn on the upper arm for up to 15 days, with data streaming via Bluetooth to a proprietary smartphone app every minute. It is water resistant and is designed to replace most finger-based blood glucose tests.

The Medtronic Guardian 4 is best-suited for insulin pump users. It can be worn for 7 days with readings occurring every 5 min and wireless transmission to compatible devices like the MiniMed 780 G insulin pump without the need for calibration. Like the FreeStyle Libre 3, it is waterproof for up to 30 min at more than 2 m depth.

The Eversense 365 is considered the first long-term implantable CGM, since its implantable sensor can be attached for the duration of a year, whereas the transmitter is removable and rechargeable. It requires timely fingerstick calibrations; however, it is appreciated for its reliability and accuracy, and is able to generate alerts directly on the device and transmit data to the Eversense mobile app.

The Dexcom Stelo is the first over-the-counter CGM designed for type 2 diabetes, featuring a biosensor worn on the back of the upper arm for up to 15 days, performing glucose readings every 5 min and delivering them every 15 min to a smartphone app. It is not compatible with insulin pumps or other devices, and it does not feature real-time alerts, therefore making it suitable with those individuals without highly critical metabolic conditions.

#### 5.1.4. Temperature and Skin Conductance Sensors

Body temperature is used for several applications, including fever tracking, identification of ovulation and menstrual cycle, baby temperature monitoring to prevent sudden death, and others. Traditional, mercury-based thermometers have been progressively replaced by digital solutions, which are accurate alternatives that do not use potentially dangerous pollutants and have gained momentum especially since the COVID-19 pandemic. Nevertheless, wearables for temperature tracking also exist, with different complexities and indications for usage.

Some of the most popular solutions in this regard include the following:

- Core 2 (greenTEG AG, Rümlang, Switzerland) [126];
- AS6221 (OSRAM Licht AG, Munich, Germany) [127];
- E4 Empatica (Empatica Inc., Cambridge, MA, USA) [128];
- Tempdrop armband (Tempdrop, Tel Aviv, Israel) [129].

The first three solutions are conceived for extensive use across nearly any kind of application, whereas the Tempdrop alternative, despite being flexible for different uses, is specifically designed for identifying the fertile window based on temperature with a related, purposely designed smartphone app, and is therefore less suitable for the use case scenarios described in the present paper.

Skin conductance is a very important parameter to be monitored in reference to the activation of the Autonomic Nervous System (ANS), particularly regarding its sympathetic branch. Different solutions have also been implemented in this regard, such as the following:

- Shimmer3R GSR+ (Shimmer Sensing, Dublin, Republic of Ireland) [130];
- E4 Empatica (Empatica Inc., Cambridge, MA, USA) [128].

Shimmer solution is specifically designed only for the skin conductance signal, with the possibility to capture an Optical Pulse/Photoplethysmogram (PPG) and a proprietary user interface running under Windows or Android OS, whereas the Empatica solution, already mentioned above, is intended to provide multiparametric monitoring of health status through a smartband, featuring an excellent wearability.

#### 5.1.5. Ingestible Biosensors

Undoubtedly, ingestible biosensors represent the continuous line between the present edge solutions in sensing and the future of personalized medicine, with promising solutions emerging from research centers daily. Smart pills are composed of sensors and wireless technology, allowing the measurements of various physiological parameters, including temperature, pH [131], and glucose levels [132], or even enabling a preliminary diagnosis of gastrointestinal diseases [133], streaming data in real-time outside the body to a repository or a purposely designed app. Such systems are designed to navigate actively (the movement is conveyed by specific ligands functionalized on the surface of the pill or through an external magnet) or passively (following the gastroesophageal tract and the peristaltic movements) through the digestive system [134].

#### 5.1.6. Environmental Sensors

**Figure 2:**
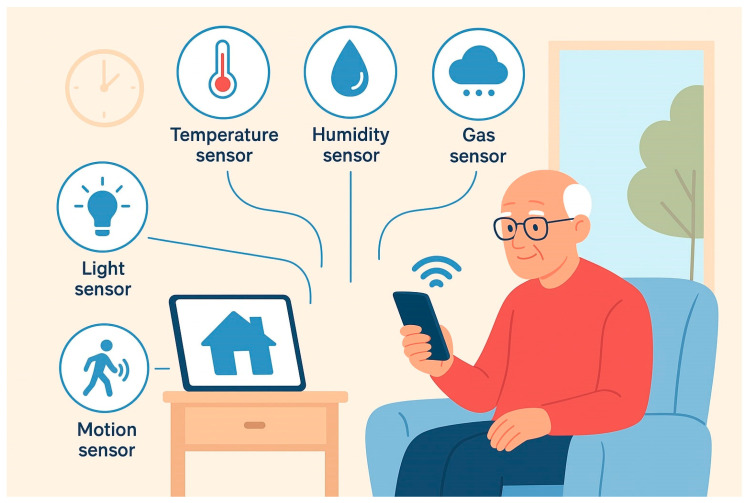
Simple scenario for environmental monitoring (image generated with ChatGPT (GPT-5 (free version)) Generative AI tool).

Environmental sensors include a wide range of solutions for a plethora of applications. For the present review, priority has been given to these solutions, which are capable of providing information related to the home environment and constitute the core of smart home applications. These include the following (see Figure 2):

- Temperature sensors to monitor thermal conditions at home and eventually intervene in extreme cases of climate issues that are potentially harmful for the individual’s health.
- Humidity sensors to measure the amount of water vapor in the air to be combined with temperature sensors for complete climate monitoring.
- Gas sensors capable of detecting different gases, including carbon monoxide, volatile organic compounds (VOCs), smoke, etc., in home environments, possibly triggering alarms in case certain thresholds are reached or exceeded.
- Light sensors to measure ambient light levels to optimally manage automated lighting and smart home applications.
- Motion sensors, like those relying on Passive InfraRed (PIR) technology, to build up security systems for anti-intrusion or environmental monitoring.

Among the commercial solutions embedding multiple of these categories, one of the most popular ones is the Airthings View Plus 2860 (Airthings, Oslo, Norway), capable of detecting various pollutants including Particulate Matter (PM1, PM2.5), VOCs, CO_2_, Radon, and others beyond just temperature and humidity, and is particularly suitable for indoor measurements [135].

### 5.2. Applications for Nutritional Personalization

Wearable devices are particularly suitable for linking time frames of feeding to related physiological responses, including fluctuations of glycemic levels after sugar consumption [136], the identification of specific response patterns [137], and the assessment of the adherence to specific health-related recommendations, including those around physical activity and sleep [138]. Taken together, such information can be useful to define personalized plans for nutrient assumption to ultimately meet metabolic goals with the aim of improving the overall quality of life in elderly individuals [136,139,140,141].

### 5.3. Limitations and Challenges

When considering the actual framework of wearable devices, the technological scenario experiences a continuous evolution, with novel devices and functionalities released daily or weekly by the most popular manufacturers. As time passes, performances of the selected devices grow, limiting the current drawbacks of most devices and enabling an improvement in cost affordability [142]. Nevertheless, the main challenges in this regard include device accuracy (i.e., mainly due to motion artifacts affecting the measurements of sensors, like optical ones, inconsistent fit dealing with sensor contact, or intrinsic algorithm flaws for different users and activity types), the variability between different devices, signal noise, battery duration, data interoperability between parties, as well as the translation of raw signals into useful, effective, and reliable nutritional guidance, which should necessarily involve medical specialists [143,144]. The theme of usability at large is another key aspect to be taken into account. This might refer either to the ease of use of such devices for the different categories of end-users depending on their age and, in most cases, technical and technological knowledge, or to the fact that many algorithms that translate the raw data into information and guidance are tailored more on the younger cohorts, which, in some cases, makes their generalizability to the elderly an open issue [145,146,147,148]. However, it is reasonable to forecast their improvements in this regard as long as they are being used by the largest and most heterogeneous groups of individuals possible.

## 6. Sensory and Physiological Data: Toward an Integrative Model

### 6.1. Integration Background

Sensory perception shapes the motivational aspects of eating behavior [149,150]. This refers to the way that how an individual perceives the sensory characteristics of food, including its taste, aroma, and texture, strongly influences eating episodes, notably in their initiation, prosecution, and termination [151]. At the same time, physiological signals, such as glucose dynamics, hormonal responses, and autonomic features, reflect the metabolic needs of the body and its responses to nutritional intake [152,153,154]. Until recent decades, these two parts have been analyzed separately and by different professionals, with sensory sciences focusing on subjective experiences and food preferences, and physiological sciences studying energy balance and metabolic control. However, their full integration can lead to the definition of a comprehensive, person-centered model of eating behavior to capture the hedonic and motivational factors of food choice and consumption and to understand the underlying physiological mechanisms and outcomes preceding and following eating events [155]. Such a view enables the understanding and interpretation of eating not just as a response to hunger but as a fully dynamic interaction between sensory pleasure, metabolic regulation, and behavioral contexts, from a fully holistic perspective.

### 6.2. The Integrated Sensory–Physiological System: Practical Steps and Applications

The real integration of the two domains would combine sensory assessments with continuous physiological monitoring and the analysis of adaptive data. In this, some basic components can be recognized:

- Basal sensory profiling. A standardized assessment is necessary to draw both the individual’s sensory profile in terms of taste, smell, and texture, and hedonics, in terms of responsiveness and sensitivity. This may serve as a reference for individualized interventions.
- Physiological monitoring. Through wearables, on all parts of the body previously indicated, it is possible to detect and record the body metabolic state to precisely characterize the physiological effects brought by specific sensory stimulations or the consumption of specific meals on the individual.
- Event-based sensory logging. User-friendly interfaces can allow users to record their sensory experiences, during or after sensory experiences or events in order to minimize recall biases and provide useful contextual information about the experience or the food consumption.
- Data analysis. Intelligent algorithms and models can integrate subjective sensory reports and implicit, objective physiological data to discover patterns and relationships such as, for example, those existing between sensory properties and personal feelings for satiety or other transient conditions. In such a framework, AI has been employed to suggest food and beverage pairings based on flavor profiles, enhancing the overall dining or drinking experience [156,157], and this approach probably represents the next big revolution in the field.
- Adaptive feedback. Analyses can be transformed into feedback in real-time or near-real-time, with the possibility to become real personalized recommendations for all consumers, for example, to optimize meal timing and ingredients and adapt these to the specificities of the individual.

This overall process might find its application in a plethora of use cases, including the compensation of sensory decline in older adults in the framework of undernutrition/malnutrition prevention, the reduction in post-prandial glycemic peaks with the maintenance of sensory qualities and food palatability, and the optimal management of behavioral fluctuations related to nutrition with respect to the current physiological and nutritional state and demands of an individual.

### 6.3. Artificial Intelligence and Digital Twin

Artificial Intelligence (AI) and particularly Machine Learning (ML) represent the present and the future of global trends in biomedical research and beyond that offer unprecedented possibilities and highly powerful tools for the identification and modeling of complex, individual relationships among sensory features, meal composition, and physiological outcomes [158]. Focusing on ML, both supervised and unsupervised models can offer the possibility to uncover latent patterns otherwise difficult to be detected using traditional statistics. Examples in this regard include the prediction of which sensory cues are most effective in promoting satiety in a specific individual or the offering of personalized nutritional guidance based on meal logs and blood glucose levels [159].

In this regard, the concept of the Digital Twin appears to be pivotal. The Digital Twin is a virtual representation of a physical object, system, or process that is used to simulate, analyze, and optimize its real-world counterpart through real-time data and advanced algorithms [160]. The model is capable of simulating the possible effects of hypothetical dietary changes before its actual implementation, therefore serving as a personalized experimental platform and enabling the precise optimization of dietary interventions that are both pleasurable and physiologically beneficial with no risk for the “real” individual or patient; this is fostering its application in the framework of the improvement of decision making and treatment selection [161].

## 7. Applications and Perspectives for Healthy Aging

### 7.1. Nutritional Personalization and Clinical Targets

The integration of sensory and physiological/behavioral data provides a novel opportunity to advance nutritional personalization and to improve clinical outcomes. The complex interaction between reduced appetite, sensory decline, and altered metabolic efficiency gives rise to several age-related conditions, including undernutrition, sarcopenia, and metabolic dysregulation, which are real threats for current clinical systems. Therefore, the combination of physiological feedback and individual sensory preferences would lead to tailored, person-centric, precise, appropriate, and cost- and outcome-efficient interventions. To this end, nutrient-enriched, sensory-appealing foods can be introduced to maintain health in individuals at risk, such as lean body mass in subjects at risk of sarcopenia, in addition to being possibly coupled with the monitoring of real-time glycemic and activity levels [162]. Also, personalized nutrition advice, informing the end-user about available edible compounds with suitable beneficial characteristics, can be issued via consumer technologies. Sensory and metabolic responses can support clinicians in the search for dietary personalization to maximize both the adherence and efficacy of nutritional treatment, with the possibility of continuous adaptation to changeable physiological conditions and sensory abilities.

**Figure 3:**
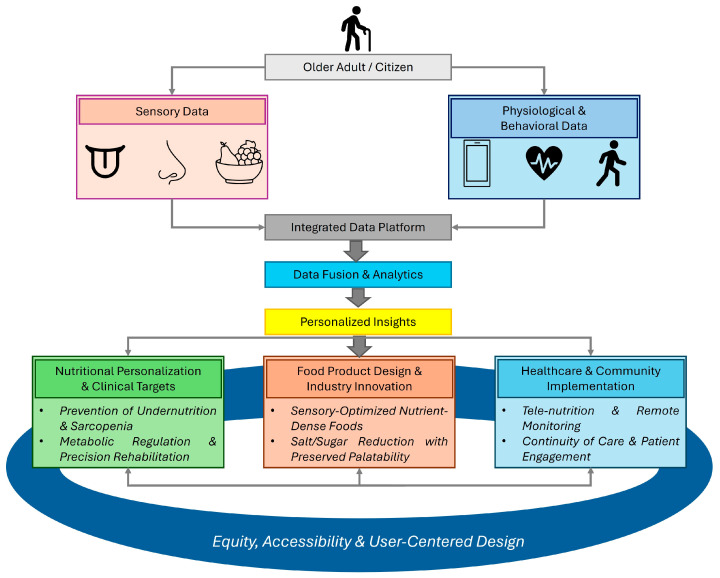
Integrative framework for older adults’ sensory, physiological, and behavioral data with respect to nutritional aspects.

Overall, this can be translated into the early detection of food-related conditions, like malnutrition in individuals at risk, enabling responsive, efficient dietary adjustments, and informing precision rehabilitation strategies. In the long-term, such systems may contribute to the maintenance of functional independence and quality of life of aging populations, bridging the gap between nutritional science, sensory health, and digital medicine (see Figure 3).

### 7.2. Food Product Design and Industry Implications

The food industry would largely benefit from the insights derived from sensory science and sensory integration with physiological and metabolic data [163,164]. Traditional product design often prioritizes hedonic appeal or nutritional labeling alone; however, their integration would enable the creation of novel, functionally and sensorially optimized foods [165]. For example, food industries can develop highly nutritious snacks reformulated to match the specific sensory needs of older adults, like soft textures for those with mastication and swallowing difficulties [166]. At the same time, novel foods that are reduced in salt or sugar could preserve their palatability through the enhancement of aroma or cross-modal sensory compensation, without compromising health targets [167].

Furthermore, as recently discussed by Shariati et al. [24], the rapidly aging global population represents a growing and heterogeneous consumer group with specific nutritional and sensory needs. Age-related changes in sensory perception, combined with increased dependency and health issues, also in terms of the ability to explicitly state their own feelings, significantly influence food preferences and acceptance in elderly individuals. Consequently, sensory evaluation methods developed for younger adults cannot be directly transferred to older populations, underscoring the need for age-adapted approaches to accurately assess food perception and support the development of suitable products.

In this context, real-time feedback from elderly consumers derived from wearables, collected both in a structured (i.e., the panel) or non-structured (i.e., during a meal) environment, could guide product development in an iterative manner to test formulations and demonstrate the related metabolic benefits, eventually supporting regulatory and marketing innovation and positioning sensory–physiological optimization as a quality standard in healthy aging nutrition.

Overall, from a broader perspective, integrating these insights into food product design may strengthen the connection between the agri-food sector and healthcare systems, fostering collaborative ecosystems that promote both pleasure and prevention in dietary behavior, with the individual becoming pivotal in the whole food product design [168].

### 7.3. Implementation in Healthcare and Community Settings

An effective implementation of integrated sensory–physiological systems requires a thorough translation into practical frameworks for healthcare and community nutritional programs. Nonetheless, in clinics, such approaches can have the ability to complement the classical dietetic assessment through the continuous assessment of pivotal indicators, including glycemic and heart rate variability, as well as the individual’s self-reported satisfaction, to guide personalized adjustments to meal plans or the eventual supplementation strategies, where applicable [158].

As such, community-based programs and institutional meal services, such as nursing homes, rehabilitation centers, and so forth, could use a similar approach to optimize menu design and real-time track the outcomes over time. In that, tele-nutrition platforms play a central role: through the combination between sensory assessment and wearable data, clinicians can receive and provide continuous feedback and adaptive guidance without requiring the patients to undergo frequent visits at clinics, which is particularly impactful for the aging population for which such an approach appears to be the most sustainable and accessible, diminishing the drop-out likelihood and the need for vehicular displacement and also ultimately helping the surrounding environment. In addition, automatization in data collection within a unique repository, such as a uniform electronic health record [169], would enable the implementation of the continuity of care, facilitating communications among the different health professionals and, in some instances, creating guidance for the patients themselves [170] to foster active citizen participation to their health status maintenance.

### 7.4. Equity, Accessibility, and User-Centered Design

The integrated nutrition systems need to present some key features in order to be successfully adopted by the end-users they are designed for, including accessibility and inclusivity. In this, user-centered design appears to be pivotal, with key priorities paid to simplicity, intuitiveness, and personalization of the user experience. User interfaces should then minimize the cognitive load for the elderly, with clear visual and auditory feedback, large fonts, and minimal navigation steps, and good affordability to avoid the so-called “digital divide”; this would, therefore, enable all citizens, independent of their economic status, to access technological solutions. Reducing the digital divide would also require community-based digital training initiatives, public–private partnerships, and cooperation with social service organizations. Also, co-design should be fostered involving end-users, clinicians, and caregivers to further enhance acceptance and long-term adherence to digital therapies. Finally, linguistic adaptation should be implemented to guide content design while considering on the cross-cultural relevance of items across populations [171,172].

### 7.5. Privacy and Data Protection

The overall framework described in the present paper necessarily involves the active engagement of elderly people and their related personal data acquisition, storage, and processing, therefore suggesting the need for a proper data management plan to maintain privacy and to protect data. In fact, all in all, nearly every digital interaction generates health-relevant information, allowing scientists to identify disease trajectories based on the online activity of the users [173,174,175,176,177]. Around the globe, several regulations exist, like the EU GDPR 2016/679 regulation which came into force in 2018 and focuses on employment, public access, research, national identity processing, and defining how data is collected, stored, used, and transferred, with particular emphasis on transparency and control. On the other side of the Atlantic, the US has adopted a more sector-specific approach with different protection levels deployed according to the Health Insurance Portability and Accountability Act (HIPAA) [178,179], with genetic information processing falling under the Genetic Information Nondiscrimination Act (GINA) [180], although several improvements should be made to further preserve the integrity of data and the digital privacy of the individuals whose data are being processed. This topic represents one of the main, trans-disciplinary fields of improvement in the broad framework of digital and consumer technologies which also affect aging and its related personalized nutrition.

## 8. Conclusions and Future Directions

As previously discussed [181], each technique used for food quality assessment presents specific strengths and limitations that must be carefully evaluated according to the application context and the technological objectives. This consideration becomes particularly relevant when addressing elderly populations, in whom sensory impairment and cognitive decline may significantly affect the reliability of conventional evaluation methods [24].

Sensory panel testing remains a central tool in food quality assessment [182], especially due to advances in statistical data analysis. Its main strength lies in providing a comprehensive and reliable description of a product’s sensory attributes and overall quality. However, the limited feasibility of engaging elderly individuals in sensory training substantially constrains the use of trained panels composed of older assessors. In parallel, reduced social participation and mobility further limit the involvement of elderly individuals in consumer studies.

When food characterization relies on human perception, emotions elicited by food play a critical role and should be considered wherever possible. Emotional assessment remains methodologically challenging, but implicit approaches appear less prone to judgment-related bias than explicit self-reporting methods [181].

Overall, sensory perception and food choice are driven by complex cognitive processes shaped by individual physiological characteristics and psychological factors, including context, socio-cultural background, prior experiences, and emotional state.

From this perspective, integrating intrinsic sensory approaches with extrinsic physiological measurements, relying on updated, more accurate and reliable, yet affordable and therefore widespread devices, may represent a promising strategy to balance data accuracy and feasibility, while enabling the partial validation of subjective perceptions through objective responses. The combination of standardized sensory science protocols tailored for elderly consumers with wearable-based physiological monitoring could open new avenues for personalized nutritional care in aging populations, especially with the growing availability of related data, having the potential to enable the effective application of AI approaches aiming at the personalization of nutritional support. Aligning dietary interventions with individual sensory profiles and metabolic states has the potential to improve adherence, maintain nutritional adequacy, and enhance quality of life.

Despite this potential, evidence from studies explicitly designed to evaluate integrated approaches involving elderly populations—and to compare them with single-method strategies—remains scarce. Future research should address this gap, adopting synergistic and ethically sound methodological frameworks to overcome the limitations of current sensory evaluation studies in aging individuals.

## Figures and Tables

**Table 1:** Sensory evaluation methods in the elderly (CATA: Check-All-That-Apply; IDDSI: International Dysphagia Diet Standardisation Initiative; TDS: Temporal Dominance of Sensations) (modified from [24]).

Main Goal	Age-Related Sensory Issues	Recommended Sensory Methods	Strengths	Limitations	Key References
Age-related food preferences	Decline in olfactory and gustatory sensitivity; selective loss of complex aromas; reduced salt, sour, bitter, and umami perception	Simplified hedonic scales; adapted descriptive analysis; aroma-enhanced testing	Allows identification of sensory shifts associated with aging; supports targeted reformulation	High inter-individual variability; sensory losses often unperceived by assessors	[82,83,84]
Dietary preferences	Reduced appetite; texture sensitivity; chewing and swallowing difficulties; dysphagia	Hedonic testing; texture-focused sensory evaluation; IDDSI-compliant assessments	Directly links sensory properties to nutritional intake and acceptability	Preferences strongly influenced by health status, autonomy, and living conditions	[85,86,87]
Medication effects on sensory perception	Polypharmacy-related taste and smell disorders; oral dryness; depression-related sensory impairment	Pre-screening questionnaires; controlled sensory testing	Improves interpretation of sensory data by accounting for confounders	Difficult to isolate drug effects; high heterogeneity	[82,88,89]
Tests for rapid sensory profiling	Cognitive fatigue; reduced attention span; sensory decline	CATA; TDS; ranking-based methods	Low cognitive demand; suitable for elderly with sensory and cognitive limitations	Reduced sensory detail compared to full descriptive analysis	[90,91,92]
Practical considerations for experimental setup	Decline in memory, attention, and processing speed; fatigue	Visual or auditory scales; assisted testing; reduced sample numbers	Enhances feasibility, comfort, and compliance	Increased testing time and costs	[93,94]
Optimal assessment strategies	Sensory and cognitive heterogeneity; technological barriers	Hybrid approaches (trained panels + elderly consumers); simplified protocols	Balances methodological rigor and ecological validity	Requires tailored logistics and trained personnel	[93,95]

**Table 2:** Comparison between mostly purchased smartwatches (as of 20 October 2025 on the European market—conversion rate: EUR 1 = USD 1.16) (ECG: electrocardiogram; SpO_2_: oxygen saturation).

Biomedical Signal(s)	Device			
	Apple Watch Series 8 (EUR 297)	Samsung Galaxy Watch 5 Pro (EUR 195)	Amazfit GTR 3 Pro (EUR 99)	Fitbit Versa 4 (EUR 195)
ECG	Yes	Yes	No	No
Heart Rate	Yes	Yes	Yes	Yes
SpO_2_	Yes	Yes	Yes	Yes
Inertials	Yes	Yes	Yes	Yes
Physical activity	Yes	Yes	Yes	Yes
Stress detection	Partially	Yes	Yes	Yes
Sleep monitoring	Yes	Yes	Yes	Yes
Fall detection	Yes	Yes	Not declared	No
Respiration	Yes	Yes	Yes	Yes
Skin bioimpedance	Not declared	Yes	No	No

## Data Availability

No new data were created or analyzed in this study.

## Abbreviations

The following abbreviations are used in this manuscript:

AI	Artificial Intelligence
ANS	Autonomic Nervous System
B2C	Business-to-Client
BLE	Bluetooth Low Energy
CATA	Check-all-that-apply
CGM	Continuous glucose monitoring
HR	Heart Rate
HRV	Heart rate variability
IDDSI	International Dysphagia Diet Standardisation Initiative
ML	Machine Learning
PIR	Passive InfraRed
PM	Particulate Matter
PPG	Photoplethysmogram
SaaS	Software-as-a-Service
SpO_2_	Oxygen Saturation
TDS	Temporal Dominance of Sensations
VOC	Volatile organic compounds
